# LINCking the Nuclear Envelope to Sperm Architecture

**DOI:** 10.3390/genes12050658

**Published:** 2021-04-27

**Authors:** Francesco Manfrevola, Florian Guillou, Silvia Fasano, Riccardo Pierantoni, Rosanna Chianese

**Affiliations:** 1 Dipartimento di Medicina Sperimentale, Università degli Studi della Campania L. Vanvitelli, Via Costantinopoli 16, 80138 Napoli, Italy; francesco.manfrevola@unicampania.it (F.M.); silvia.fasano@unicampania.it (S.F.); riccardo.pierantoni@unicampania.it (R.P.); 2 PRC, CNRS, IFCE, INRAE, University of Tours, 37380 Nouzilly, France; florian.guillou@inrae.fr

**Keywords:** LINC complex, SUN, KASH, actin, spermatozoa, male fertility

## Abstract

Nuclear architecture undergoes an extensive remodeling during spermatogenesis, especially at levels of spermatocytes (SPC) and spermatids (SPT). Interestingly, typical events of spermiogenesis, such as nuclear elongation, acrosome biogenesis, and flagellum formation, need a functional cooperation between proteins of the nuclear envelope and acroplaxome/manchette structures. In addition, nuclear envelope plays a key role in chromosome distribution. In this scenario, special attention has been focused on the LINC (linker of nucleoskeleton and cytoskeleton) complex, a nuclear envelope-bridge structure involved in the connection of the nucleoskeleton to the cytoskeleton, governing mechanotransduction. It includes two integral proteins: KASH- and SUN-domain proteins, on the outer (ONM) and inner (INM) nuclear membrane, respectively. The LINC complex is involved in several functions fundamental to the correct development of sperm cells such as head formation and head to tail connection, and, therefore, it seems to be important in determining male fertility. This review provides a global overview of the main LINC complex components, with a special attention to their subcellular localization in sperm cells, their roles in the regulation of sperm morphological maturation, and, lastly, LINC complex alterations associated to male infertility.

## 1. Introduction

Spermatogenesis is a differentiation process, spatio-temporally coordinated, responsible for spermatozoa (SPZ) production [1,2]. During spermatogenesis, male germ cells undergo three fundamental phases: (i) self-renewal of spermatogonial stem cells (SSC) and proliferation of spermatogonia (SPG); (ii) meiosis of spermatocytes (SPC) for haploid round spermatid (rSPT) production; and (iii) morphogenesis of rSPT into SPZ [3,4]. During these events, the nuclear structure undergoes a dynamic remodeling to favor the meiotic pairing of the homologous chromosomes, their separation to form haploid cells, and, finally, the extreme chromatin condensation in order to protect the paternal genome from chemical and physical insults and to reduce sperm head size, thus guaranteeing the hydrodynamic shape of SPZ [5,6,7]. Additionally, during spermiogenesis, rSPT undergo massive morphological changes including acrosome and tail formation, cytoplasm elimination, and nuclear condensation that requires histone replacement by protamines (PRMs) [3,6,8,9]. 

In this regard, the profound rearrangement of the nuclear architecture deeply involves an extensive remodeling of the nuclear envelope (NE), including the composition and the localization of its components. Outer (ONM) and inner (INM) nuclear membranes, periodically fused at sites forming the nuclear pore complexes (NPCs), play different roles: ONM proteins are especially implicated in nuclear positioning and movement, while INM proteins are in close association with nuclear lamina and chromatin, thus regulating a wide range of nuclear functions such as DNA replication and transcription, chromatin organization, mitosis, and meiosis [10,11,12,13]. 

During spermatogenesis, nuclear remodeling particularly concerns SPC and SPT. During meiosis, NE proteins, in collaboration with cytoskeletal elements, govern chromosome spatial arrangement [14,15]. Both the reorganization of the nuclear lamina and the cytoplasmic microtubule-associated motor forces drive chromosome movements [14,15]. Beyond its action during male meiosis, NE acts as a platform for several structural changes, typical of the spermiogenesis phase, such as nuclear elongation, acrosome biogenesis, and flagellum formation [16,17,18,19]. Thus, several NE proteins cooperate with acroplaxome and manchette structures in order to orchestrate nuclear reshaping [19,20].

In cell nuclei, chromosomes are finely organized and located in specific nuclear regions named chromosomes territories [21,22]. Interestingly, sperm chromosomes also do not have a random localization in the nucleus; indeed, a specific longitudinal or radial positioning of them seems to be involved in the accurate paternal gene expression pattern after oocyte fertilization [23,24,25,26,27]. NE and its integral membrane proteins play a central role in chromosome positioning as well as in sperm head formation [16,28,29].

The linker of nucleoskeleton and cytoskeleton (LINC) complex is an NE-bridge structure involved in the connection of nucleoskeleton to the cytoskeleton, crucial for nuclear migration, positioning, and anchoring [19,30,31]. The LINC complex is formed by two transmembrane protein families, both having a single-transmembrane-pass: Nuclear Envelope Spectrin repeat proteins (NESPRIN), located in the ONM, and Sad1p-UNC84 (SUN) domain proteins, located in the INM [32,33,34]. 

Nesprins have a central region of variable length and a C-terminal Klarsicht/ANC-1/Syne homology (KASH) transmembrane domain that facilities NE localization [35]; their N-terminal domain is bound to the cytoskeleton [36,37,38]. SUN proteins are, instead, characterized by a nucleoplasmic N-terminal domain bound to nucleoskeleton and a C-terminal domain that is projected into the perinuclear space (PNS) [15].

The nesprin family includes four members, also identified as KASH proteins, encoded by four genes, known as SYNE1-4; the SUN family includes five members (SUN1-5) [36,39,40]. The largest isoforms of the nesprin family are KASH1 and KASH2, the only isoforms able to directly contact the filamentous actin (F-actin); KASH3 associates with intermediate filaments by using a plectin-binding motif [41]. Instead, KASH4 interacts with kif5b, a subunit of the microtubule motor kinesin-1, and therefore indirectly with microtubules [42,43]. In addition to these four members, KASH5 appears the most divergent; its N-terminus interacts with dynein–dynactin, which mediates microtubule binding, thus driving rapid chromosomal movements that facilitate homologous chromosome pairing [44]. In doing so, KASH5 has a key role in meiosis, and it is essential for fertility. Among SUN proteins, SUN1 and 2 are ubiquitously produced in somatic cells; instead, SUN3, 4 (whose alternative name is SPAG4), and 5 (whose alternative name is SPAG4L) are restricted to the testis [32,39,45].

Functions of cytoplasm and nucleus are tightly coupled not only through the LINC complexes, but also through NPCs that are intimately associated [46]. First of all, the final destination of SUN proteins on the INM needs the protein trafficking across the NPCs. Furthermore, SUN1 co-localizes with nucleoporin 153 (Nup153) and affects the distribution of NPCs [47]. SUN1, in combination with SUN2, interacts with messenger ribonucleoprotein particle components, thus having a direct role in mRNA export through the NPC in mammalian cells [48].

A regulatory action of the LINC complex during spermatogenesis and sperm maturation is well reported. The LINC complex participates in meiotic recombination supporting a proper chromosomes pairing, as well as telomere movement and attachment; in fact, genetic disruption of Sun1 in mice prevents telomere attachment to the nuclear envelope, efficient homolog pairing, and synapsis formation in meiosis [49]. In addition, KASH5, a specific partner of SUN1, actively participates in this event [50]. SUN2 also interacts with KASH5, forming the complex SUN1/SUN2/KASH5 involved in the tethering of meiotic telomeres to the NE [51,52]. Other SUN proteins involved in meiotic recombination include SUN5. This function is strictly interconnected to nuclear lamina, a meshwork of intermediate filament proteins located in the nucleoplasm, between INM and chromatin [53]. In mammals, A-type lamins include A, C, and a male meiosis-specific isoform C2; B-type lamins include B1, B2, and a spermatid-specific isoform B3. In mouse, SUN5 co-localizes with lamin B1 and is involved in NE reconstitution and nuclear migration occurring during meiotic division [54]. In agreement, the SUN5/KASH2 LINC complex may be involved in mouse SPC meiotic division [55]. LINC complex has also a consolidated role in mammalian sperm head formation and head to tail connection, and, therefore, it seems to be important in determining male infertility [45,56,57]. 

In this scenario, focusing on sperm cells, we report a global overview of the main LINC complex components and their regulatory action in mechanotransduction, with a special attention paid to their subcellular localization in SPZ, their roles in the regulation of sperm morphological maturation, and, lastly, LINC complex alterations associated to male infertility.

## 2. The Role of the LINC Complex in Mechanotransduction

Mechanobiology evaluates how mechanical forces impact on cell morphology and physiology. Mechanical forces have great relevance in cell differentiation, development, and several disease states. Cells respond to these forces by activating molecular mechanisms in their microenvironment. Most mechanical processes lead to nuclear movement, shape, and positioning, as well as chromatin folding and gene expression, in which the LINC complex plays a critical role.

**Figure 1:**
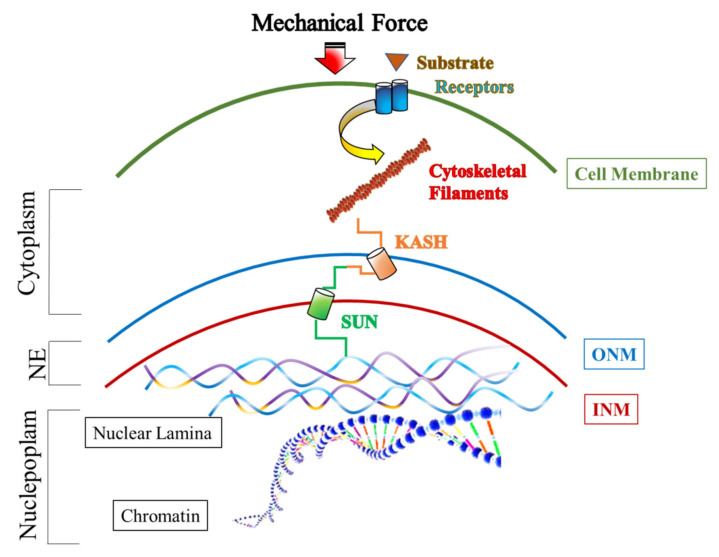
Schematic representation of mechanical force transduction from the cell membrane to the nucleus. Following mechanical stimulus and activation of membrane receptors, a signal transduction pathway is triggered to cytoskeletal filaments that interact with LINC complex components. These last, in turn, interact with nuclear lamina that transmits mechanical force to the chromatin. Finally, chromatin folding changes, induced by mechanical force transduction, modulate gene expression.

Mechanotransduction is just the route by which cells transform extracellular mechanical signals into biochemical signals, involving cytoskeletal–nucleoskeletal connections; in other words, extracellular forces, generated from the matrix, through the propagation of mechanical signals along the cytoskeleton, regulate NE composition, acting on the LINC complex, nuclear shape, chromatin organization, and gene expression, as a final effect (Figure 1) [58,59,60]. The idea that the LINC complex may act as a force-sensitive-signaling hub in order to fine-tune nuclear responses came from studies on isolated nuclei, subjected to physical forces [61]. Studies on this aspect demonstrate, in fact, that perturbations of the LINC complex or disruption of the interaction between the LINC complex and nuclear lamins cause cytoskeletal disorganization, impair the cytoskeletal force transmission to the nucleus, and alter gene expression [62]. 

One of the main cytoskeletal actors in the mechanotransduction pathway is the actin. Actin plays a crucial role in different cellular functions such as (i) cell motility, (ii) cell signaling, and (iii) cellular response to mechanical stimuli. Recent findings show the newest actin functions, highlighting how the actin structures around NE may be a key factor in the regulation of nucleus movement as well as gene expression [63]. In this scenario, the LINC complex mechanically connects the nucleus to the cytoskeletal actin, which in turn mediates nuclear movement and nucleus position in cells [32]. The actin-to-LINC complex connection is mediated by KASH proteins, which, on one side, interact with SUN partner at the NE level, while on the other side they interact with cytoskeletal actin through a specific actin-binding domain. The actin role in nucleus movement via the LINC complex is well described. Indeed, KASH2 and SUN1/2 alterations negatively impair nuclear migration during mammalian retinal development [64]. In addition, prior cell migration and transverse actin filaments interact with KASH2 to form transmembrane actin-associated nuclear (TAN) lines, which provide a retrograde flow useful for nucleus movement behind the centrosome. Interestingly, depending on the SUN partner, two different nuclear movements occur. In detail, KASH2/SUN2 interactions induce rearward movement via TAN lines, while KASH2/SUN1 interactions induce toward movement via microtubules [65,66,67,68].

Around the nucleus, actin also forms a filamentous structure covering the nuclear surface named perinuclear actin CAP, that, interacting with KASH2 or KASH3, participates in nucleus orientation, nuclear shape, and finally mechanotransduction, protecting the nucleus from mechanical deformation [69,70,71]. KASH2/3 depletion is associated with the inhibition of perinuclear actin CAP assembly and negatively affects mechanotransduction [72].

One of the kinds of main intracellular mechanosensor signaling generated from mechanical cues and linked to actin stimulation is the Hippo kinase signaling cascade. This evolutionarily conserved pathway, involved in the regulation of cell proliferation and fate, tissue differentiation, and organ size, is triggered downstream from a mechanical force able to induce actin activation [73,74,75]. It also includes Yes-associated protein (YAP) and its homologous protein Transcriptional Co-Activator With PDZ-Binding Motif (TAZ), two transcriptional co-activators able to deliver mechanical cues to the transcriptional machinery of the nucleus by shuttling between the cytoplasm and nucleus [76,77,78].

Actin–LINC complex interaction plays a key role in mitosis and meiosis. In the first case, the actin–LINC complex regulates centrosome positioning and ensures that no chromosome mis-location occurs, while during meiosis actin polymerization regulates the nuclear movement [79,80]. Finally, the actin–LINC complex regulates gene expression. In several cell types, actin contractility regulates nuclear volume and, mechanically, affects chromatin conformation and accessibility to transcriptional factors, with consequent alteration of gene expression [81]. In addition, alteration of gene expression may depend on anomalous LINC complex dependent mechanisms, as observed in the case of KASH2 downregulation that associates with alterations of gene expression in epithelial cells [82]. In other words, transcriptional regulation may depend on the LINC complex through non-mechanical mechanisms, such as changes in signaling pathways putatively mediated by this complex.

Whether the LINC complex directly contacts chromatin or establishes indirect contacts via nuclear lamina is not fully elucidated. Such a possibility is more intriguing in post-meiotic germ cells that undergo chromatin condensation through the replacement of nuclear histones with protamines [4]. In somatic cells, the Lamin B receptor (LBR), an integral protein of the INM, binds to lamin B1 and links the nuclear lamina to the chromatin through an interaction with the HP1-type heterochromatin proteins and the Barrier-to-autointegration factor (BAF) [83,84]. In germ cells, LBR is also able to temporally associate with protamine 1. In detail, during mammalian spermiogenesis, protamine 1 is highly phosphorylated to be deposited into SPT chromatin and almost completely dephosphorylated during sperm maturation [85,86]. Therefore, in elongating spermatids (eSPT), this phosphorylation is required for the temporal association of P1 protamine with LBR [87]. Known somatic lamina–chromatin interface regulators, such as lamina-associated protein LAP2ß and lamin B1, change their distribution in the SPT nucleus, concomitantly with chromosome positioning and their stabilization in specific nucleus areas [16,28,29]. Conversely, in humans, the absence of the lamina–chromatin interface in most SPT may determine the detachment of the chromatin from the nuclear lamina in order to increase its accessibility for histone–protamine exchange [84]. However, a differential expression of LBR has been demonstrated between rodents and humans, since human SPT lose a significant signal for this receptor. In addition, both BAF and its paralogue, BAF-like (BAF-L), have been observed in human SPZ. While BAF homodimers have been involved in the induction of chromatin condensation [88], BAF–BAF-L heterodimers could promote massive histone–protamine exchanges through a more open chromatin [84].

Several doubts still exist about the molecular mechanisms necessary to allow a physical and a functional interaction among the LINC complex, nuclear lamina, and chromatin, requiring more efforts to address the research in such a direction.

Beyond being a traditional component of the cytoskeleton and making contacts with the LINC complex, actin constantly shuttles between the cytoplasm and the nucleus. This dual localization is required for maximal transcription; in fact, in the nucleus, actin regulates the activity of specific transcription factors, participates in several chromatin remodeling complexes, and associates with all three RNA polymerases [89]. Intriguingly, nuclear filamentous actin (F-actin) has been found after fertilization in zygote, inside the large and decondensed pronuclei [90]. It is required for fully functional pronuclei; in fact, its perturbation causes the mis-regulation of genes related to genome integrity, generating an abnormal development of mouse embryos [90].

Other non-mutually-exclusive mechanisms of nuclear mechanotransduction, beyond LINC complex regulation, have been proposed to date. One of them concerns nuclear pore stretching. Accordingly, mechanical stimuli are able to induce physical changes of NPCs, and, therefore, mechanoactivate the NPC permeability, independently of the LINC complex [91]. In addition, mechanical forces can modulate the phosphorylation state of NE proteins through a molecular mechanism yet to be elucidated. Lamin A/C is an example of a protein whose phosphorylation modulates nuclear stiffness in response to mechanical stimulation [92]. Chromatin organization is also responsive to extracellular and cytoskeletal mechanical cues, thus controlling gene expression [60]. Perinuclear actin filaments, through the LINC complex, drive lamin A/C hyperacetylation, chromatin decondensation, and gene expression activation [93]. An alternative mechanism of gene expression regulation induced by mechanotransduction consists in the nuclear import of histone–lysine *N*-methyltransferase EZH2 and histone deacetylase (HDAC) that stimulates gene silencing through methylation patterns and gene transcription by increasing histone acetylation, respectively [94,95].

Morphogenetic movements and cell fate decisions during embryogenesis are deeply influenced by mechanotransduction. Physical compression of Drosophila embryos at the cellular blastoderm stage significantly increases the expression of the transcription factor Twist in all cells, and not only on the ventral side of the embryo, suggesting a direct link between mechanotransduction, gene expression, and embryo development [96].

## 3. Actin Functions in Sperm Head and Tail

The major cytoskeletal proteins localized in the sperm head include actin, tubulin, and spectrin that not only are implicated in the correct sperm head shaping, but also participate in capacitation and acrosome reaction (AR) [97,98,99].

Actin is the most abundant cytoskeletal protein in the sperm head; its involvement in the head structural modifications during capacitation and acrosomal exocytosis is well reported. Indeed, during capacitation, a prominent polymerization of G-actin monomers to form F-actin filament occurs in the sperm head to prevent spontaneous AR. Later, when the correct time-point for AR happens, a rapid F-actin depolymerization occurs [98,100]. While F-actin formation increases during capacitation, conversely, its depolymerization is enhanced during AR by intracellular Ca^2+^ concentration. According to these findings, the inhibition of F-actin depolymerization by phalloidin treatment associates with the loss of AR [101,102]. In details, the increase of F-actin during capacitation is dependent on the activation of Phospholipase D (PLD) and Ca^2+^/calmodulin-dependent protein kinase II (CaMKII), which modulate the increase of Phosphatidylinositol 4,5-bisphosphate (PIP2) in the sperm head [103,104]. This intricate pathway involves more players such as gelsolin and cofilin that act as negative factors on actin polymerization. Indeed, during capacitation, the binding of gelsolin to PIP2 in the sperm head promotes its tyr-438 phosphorylation by the Src kinase PKA dependent pathway, which induces an inactive form of gelsolin favoring F-actin formation [103]. Subsequently, during AR, gelsolin accumulates its active form in the sperm head to induce two parallel events: (i) F-actin depolymerization in sperm head and (ii) gelsolin reduction in sperm flagellum to allow F-actin formation, indispensable for motility increase. As gelsolin, the activation of cofilin, promotes its head accumulation, F-actin depolymerization and AR begin [105]. In addition, several actin-interacting proteins co-localize with F-actin in the sperm head, in particular spectrin and nectin-2. The first was detected in the apical and equatorial acrosomal region with an interesting re-localization on equatorial segment during the AR, suggesting a key role in the structural stabilization of this region during acrosomal biochemical changes, while nectin-2 is a crucial factor involved in sperm nuclear shaping [106,107]. 

The functional role of F-actin in sperm flagellum has been clarified by several studies in which an evident positive correlation between actin polymerization ad sperm motility has been shown. In fact, during capacitation, SPZ acquire hyperactivated motility (HAM) fundamental for a successful fertilization [108]. More interestingly, F-actin formation in sperm flagellum is an essential event for a proper HAM acquisition and is dependent on positive regulators, such as PIP2, and negative regulators, such as gelsolin and cofilin [109]. Indeed, Finkelstein and colleagues have shown that HAM development requires gelsolin and cofilin translocation in the sperm head to prevent actin depolymerization in the flagellum [110]. In addition, PIP2 reduction induces low-actin polymerization that inhibits sperm motility, confirming a positive involvement of PLD-dependent actin polymerization in HAM acquisition [109,110].

Actin polymerization seems to follow specialized structures depending on the sperm flagellum section in which it occurs. Recent studies in mouse SPZ show that F-actin forms a double helical structure in the midpiece portion that localizes with the radial center of mitochondria, following the mitochondrial sheath; instead, in the principal piece, F-actin is located in bundles from axoneme to the plasma membrane [111]. Interestingly, some actin-interacting proteins, such as spectrin and adducin, participate in flagellum integrity, elasticity, and the mitochondrial sheath reinforcement required during HAM [111,112]. In detail, spectrin and adducin co-localize with F-actin in the midpiece, while in the principal piece, adducin does not overlap with spectrin, suggesting a differential interaction pathway between these structural proteins dependent on the flagellum region in which it occurs. In addition, nectin-2 is a crucial factor involved in the recruitment of mitochondria to the middle piece of the flagellum [106]. 

## 4. A Special Focus: LINC Complex and Sperm Head

The sperm head is principally divided into two main regions: the acrosomal region and the post-acrosomal region. The nucleus, containing compacted tightly chromatin, almost completely occupies the head; it is partially covered by the acrosome, a cap-shaped organelle, originated from the Golgi apparatus and containing enzymes and receptors useful for oocyte binding and fertilization [4,113,114,115]. 

Despite differences in the shape and size of sperm head between species, some elements such as the apical acrosome, the post-acrosomal region, and the equatorial segment, are more conserved to support typical sperm head functions such as (i) AR, (ii) capacitation, and (iii) sperm–oocyte fusion.

Massive sperm head shaping is mainly driven by a cytoskeletal complex called acrosome–acroplaxome–manchette. Acroplaxome, enriched in keratin and fibrous actin, surrounds the developing acrosome and anchors it to the NE [20]. The manchette is a microtubular transient structure that appears at the beginning of the elongation phase; when the sperm head is fully formed, the manchette dissembles from NE and moves caudally to the nucleus to participate in the tail formation [116].

In female reproductive tracts, SPZ undergo the capacitation process, consisting of several biochemical modifications useful to ensure oocyte fertilization [117]. During capacitation, sperm head continues to change its cytoskeletal structure in order to induce AR [118].

In this scenario, the localization of a testis-specific isoform of SUN1, named SUN1η, is very interesting, since it has been found at the anterior pole of the nucleus facing part of the acrosomal membrane, instead of the INM [56]. There, it forms a not-nuclear LINC complex with KASH3 that, through plectin, interacts with the acroplaxome [56]. This complex also appears at the posterior part of the nucleus, excepting the implantation fossa, the site of the tail connection to the head [15].

Recent studies report SUN3 and SUN4 proteins as indispensable for sperm head formation. The expression levels of SUN3 during the first wave of spermatogenesis increase concomitantly to rSPT and elongated SPT (eSPT) accumulation. In particular, SUN3 localizes to lateral–posterior regions of spermatid nuclei, but not in the implantation fossa and in the very posterior pole of the nucleus [56]. The SUN3 partner in sperm head is KASH1, which co-localizes with SUN3 and forms a LINC complex able to facilitate the connection between the manchette and the ONM, considering its ability to bind to actin, via its actin-binding domain, or to microtubules, via dynein–dynactin complex [20,56].

**Figure 2:**
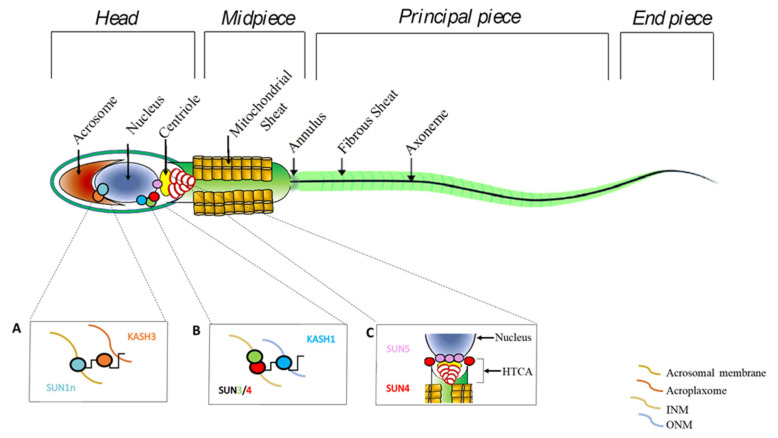
Schematic representation of principal LINC complex components in mature SPZ. (**A**) LINC complex SUN1η/KASH3 at the anterior pole of sperm head is associated with the acrosomal membrane and involved in the anchoring of acrosomal membrane to NE. (**B**) LINC complex SUN3/SUN4/KASH1 at the posterior pole of the sperm head is involved in the connection of manchette to NE. (**C**) SUN4 and SUN5 at the HTCA level in the implantation fossa are involved in the head–tail connection.

SUN4 is only expressed during spermiogenesis and specifically at the posterior pole of nucleus of rSPT and eSPT; interestingly, it is an interacting partner of SUN3-KASH1 at the posterior–lateral region adjacent to NE and in parallel to manchette, suggesting a heterotrimeric LINC complex SUN3/SUN4/KASH1 involved in the connection of manchette to NE [45]. *Sun4* knockout mice confirm the crucial role of SUN4 in sperm head formation, as it shows an aberrant number of rSPT, disrupted sperm elongation, and the loss of NE integrity, with consequent production of deformed SPZ. In addition, the lack of SUN4 induces SUN3 mis-localization in the cytoplasm and a disorganized manchette, also suggesting a function in the correct localization of SUN3 and KASH1 [45]. Figure 2 is a schematic view of the main LINC complexes in the sperm head.

In humans, SUN4 also constitutes a molecular complex with the testis-specific cytoskeletal Septin 12 (SEPT12) and the intermediate filament protein lamin B1, orchestrating sperm head formation in post-meiotic germ cells, but also tail organization [119].

Beyond actin filaments, microtubules, and intermediate filaments, the cytoskeleton also includes septins, involved in several physiological functions [120,121]. In particular, SEPT12 has been implicated in mammalian spermiogenesis; its expression has been observed around the manchette, the neck region of eSPT, and the annulus of mature sperm [119,122].

Intriguing findings concerning a hypothetical role of LINC complex in acrosome biogenesis during sperm head shaping have been reported. Frohnert et al. have characterized a novel testis-specific SPAG4L member, named SPAG4L-2, highly expressed during spermiogenesis and localized at the apical nuclear region of rSPT facing acrosomic vesicle, thus suggesting its involvement in acrosome biogenesis [20,123]. 

During nuclear elongation, SUN5 progressively migrates to the posterior pole of the nucleus in eSPT to finally reach the implantation fossa [124,125]. This localization allows the exclusion of an involvement of SUN5 in sperm head formation. Accordingly, *Sun5* knockout male mice display no defects in acrosome biogenesis, but malformations in sperm tails, as further explained.

Acrosome biogenesis also involves components of nuclear lamina. Interestingly, lamin A/C has been identified as a structural component of acroplaxome, required for acrosome biogenesis and spermatid head shaping [126]. Protein phosphorylation has been suggested as the possible regulatory mechanism influencing lamin A/C localization among the nucleoplasm, the nuclear lamina, and the cytoplasm [127]. In concert with lamin, B1 plays DPY19L2, a testis-specific INM protein, predominantly produced in SPT. Its disruption alters the subcellular localization of lamin B1 during spermiogenesis, as well as destabilizes the interaction between the NE and the acroplaxome during acrosome biogenesis. The consequence is a sperm nucleus poorly compacted, with a failure to replace histones with protamins and globozoospermia as a consequence [128,129]. 

The presence of KASH2 and KASH4 has not been detected at any post-meiotic stages of sperm development [56]. As previously described, KASH5 is the most divergent. Although it is a germ cell specific KASH protein, its localization is especially confined to the cytoplasm and, in complex with SUN1 and SUN2, it mediates telomere attachment to the cytoskeleton and chromosome movements in SPC [130].

## 5. LINC Complex and Sperm Tail

The sperm tail is a cellular structure longitudinally divided into (i) the midpiece, (ii) the principal piece, and (iii) the end piece. These sections are organized around a central microtubular structure named axoneme, consisting of a central microtubular section surrounded by peripheral microtubules and sperm tail accessory structures such as (i) outer dense fibers (ODF) that develop in the principal and midpiece, and (ii) fibrous sheaths (FS) that develop along the principal piece and mitochondrial sheaths (MS) that form a spiral array around the ODFs in the midpiece [131,132]. The tail sustains several sperm features, in particular sperm motility, a key property to ensure optimal fertilization. Flagellar movement depends on (i) a correct cytoskeleton assembly, (ii) ATP production from mitochondria, and (iii) organization of the microtubular components in the axoneme. In this regard, defects in sperm mitochondrial ultrastructure are associated with decreased sperm motility, asthenozoospermia, and, therefore, unsuccessful fertilization [133,134,135,136]. However, SPZ show a great versatility in ATP production by using both glycolysis and mitochondrial oxidative phosphorylation in sustaining tail functionality [137].

The implantation fossa, previously defined as the site of tail connection to the head, is crucial to ensure the force needed for SPZ movement. Fundamental to a proper tail attachment is the head to tail coupling apparatus (HTCA), an asymmetric structure formed during spermiogenesis, containing centrioles (proximal and distal), a dense fibrous structure named capitulum, and the segmented columns [138]. To link HTCA to the sperm head, several LINC complexes are involved. Among SUN proteins, the contribution of SUN4 and SUN5 in head-to-tail coupling is relevant, even if SUN4’s role appears inessential [57]. 

However, beyond its localization on the INM, SUN4 is engaged in the positioning of ODF proteins in the tail, acting as a link molecule between the axonemal microtubules and ODFs [39]. This evidence still appears to be a matter of debate, since the mechanism by which a classified INM protein may interact with cytoskeletal components remains to be explored.

Pasch and co-authors reported in *Sun4* knockout mice several SPZ with tails coiled around the head, suggesting an improper link between the head and the tail [45]. Yang and co-authors showed that HTCA is not compromised in the absence of SUN4, but it is less efficient in the stabilization of head/tail interaction. Indeed, HTCA appears more detached from lateral areas of the nucleus, suggesting a SUN4 role in tightening the head/tail anchorage [139]. This activity may be helped by a strong cooperation among SUN4, ODF1—the major component of ODF within the midpiece and the principal piece, SEPT12, and lamin B1. In detail, SEPT12 has been implicated in tail formation and, in concert with SUN4 and lamin B1, functionally associates at the sperm neck [122].

SUN5 is essential for head-to-tail anchoring. During spermiogenesis, SUN5 localizes in the NE, but in mature SPZ, it is strongly relocated to HTCA in the implantation fossa, underlying its crucial function in the head–tail connection [140]. In confirmation of this, eSPT of *Sun5* knockout mice show a destroyed connection between HTCA and the sperm head, with a consequent release of decapitated flagella in seminiferous tubule lumen and retention of the sperm head in the tubular epithelium [140]. A similar phonotype has been found in men with homozygous deletion or variants of *Sun5*, which produce decapitated SPZ [84]. In doing so, SUN5 may cooperate with DnaJ heat shock protein family member B13 (DNAJB13) [125], a structural component of the axoneme, in both SPT and SPZ. The molecular mechanism suggested predicts that, via DNAKB13 interaction, SUN5 prevents the separation of head from tail during the release of SPZ into the lumen of seminiferous tubules.

Figure 2 is a schematic view of the main LINC complexes in SPZ.

## 6. LINC Complex and Male Infertility

As previously described, the LINC complex shows an intricate and central role in male germ cell development. To date, more studies have reported how the loss of a proper SUN–KASH interaction leads to alterations in SPZ functions, and in this section, we summarized the latest characterized. 

The study of *Sun1* knockout male mice evidences a total infertility phenotype due to the disruption of SUN1–telomere interaction required for telomere movement during chromosome recombination in primary SPC [49,52]. As the SUN1–KASH5 complex regulates telomere movement, KASH5 alteration is also associated with inefficient telomere attachment to NE, compromising chromosome pairing [50]. Interestingly, Chi and co-authors show that *Sun1* knockout mice are reproductively infertile and molecularly show the lack of expression for coding and non-coding RNAs needed for spermatogenesis, suggesting a new role for SUN1 in gene expression regulation [141].

Regarding SUN3, it has been shown that *Sun3* knockout male mice are infertile with a damaged sperm head and tail. In detail, the major abnormalities happen during spermiogenesis, where SPT nuclei fail to elongate due to the absence of cytoskeletal force transduction to NE, fundamental for head elongation [142]. As a consequence, the animals produce a low concentration of SPZ characterized by abnormal acrosome, low motility, and globozoospermia phenotype. Pasch and coauthors demonstrate that SUN3 also participates in the connection of the manchette to NE by the heterotrimeric LINC complex SUN3/SUN4/KASH1 [45]. Accordingly, *Sun3* knockout mice show a reduction of SUN4 levels in association to abnormal manchette formation [142]. Spermatogenesis of *Sun4* knockout male mice normally progresses up to meiosis, but in the spermiogenesis phase, several post-meiotic aspects are compromised, leading to infertility. In detail, SPT nuclei are round or misshapen with abnormal chromatin remodeling. The manchette is disorganized and fails in the coupling to NE, essential for head shaping, with consequent acrosome alteration and globozoospermic SPZ production. Interestingly, in *Sun4* knockout mice, a loss of SUN3 has been observed in SPT [143]. Accordingly, Pasch et al. describe that *Sun4* knockout mice have an increased number of aberrant rSPT in the stages VIII–IX of the spermatogenic cycle, during which the typical nuclear elongation occurs. Indeed, sperm head elongation and shaping appear more compromised with consequent aberrant-shaped SPZ release [45]. Additionally, typical SUN3 distribution within the posterior NE is altered as it appears to not be located to NE, but relocated to the cytoplasm. Alterations in SUN4 content correlate to an inefficient sperm head-to-tail linkage; indeed, SPT of *Sun4* knockout mice show a detachment of the HTCA from the nuclear membrane, negatively affecting tail linkage to the nucleus [139]. Given the ability of SUN4 to interact with SEPT12, it is intriguing to analyze the reproductive phenotype of *Sept12* knockout mice that produce SPZ with morphological defects in the head and the tail, nuclear damage, and premature chromosomal condensation. Sperm from these animals causes arrested development of preimplantation embryos. *Sept12* mutations also cause teratozoospermia and oligozoospermia in humans [144,145]. 

*Sun5* knockout mice are infertile and produce acephalic SPZ. Indeed, SUN5 appears to be essential in the anchoring of the sperm tail to the head, which results in complete detachment in *Sun5* knockout eSPT [140]. This phenotype appears in accordance with several cases of male infertility due to *Sun5* gene mutations that induce SUN5 loss or variant production associated to acephalic SPZ syndrome, a form of teratozoospermia characterized by headless SPZ in the ejaculate [146,147,148].

## 7. Future Perspective 

One the most common reproductive disorders is male infertility. The elucidation of the pathological mechanisms involved is an extremely urgent need. 

The integrity of testis as well as the sperm quality may be compromised by genetic and/or epigenetic defects. In this regard, paternal exposure to a large array of factors, including endocrine-disrupting chemicals and lifestyle exposures such as stress and diet, has been suggested as a critical point for embryo development and offspring health outcomes [149]. 

Intriguingly, several intragonadal regulators, beyond hormones, orchestrate a harmonious progression of germ cells [1]. Here, we have pointed at LINC complex as an under-studied molecular system potentially involved in the regulation of important sperm physiological functions, focusing on their components and detailing their localization in sperm cells. In order to achieve competence in the LINC complex, the characterization of knockout mouse models for its main components has provided additional evidence of the biological relevance of such a complex for male fertility.

However, multiple questions still exist in this area, thus stimulating research to pursue the analysis of NE dynamics during spermatogenesis and in correlation with sperm quality, in order to identify novel potential players in sperm head shaping and tail formation. An interesting prospect may be to therapeutically target the described NE proteins, individually or in their interconnections, to recover a fertile phenotype in animal models and, in future, develop new reproductive strategies directed at infertile male patients.
